# *Deinococcus radiodurans pprI* expression enhances the radioresistance of eukaryotes

**DOI:** 10.18632/oncotarget.8137

**Published:** 2016-03-16

**Authors:** Ling Wen, Ling Yue, Yi Shi, Lili Ren, Tingting Chen, Na Li, Shuyu Zhang, Wei Yang, Zhanshan Yang

**Affiliations:** ^1^ Department of Radiation Toxicology, School of Radiological Medicine and Protection, Medical College of Soochow University, Collaborative Innovation Center of Radiation Medicine of Jiangsu Higher Education Institutions, Soochow University, Suzhou, Jiangsu, China; ^2^ Department of Radiation Genetics, School of Radiological Medicine and Protection, Medical College of Soochow University, Collaborative Innovation Center of Radiation Medicine of Jiangsu Higher Education Institutions, Soochow University, Suzhou, Jiangsu, China; ^3^ Department of Radiobiology, School of Radiological Medicine and Protection, Medical College of Soochow University, Collaborative Innovation Center of Radiation Medicine of Jiangsu Higher Education Institutions, Soochow University, Suzhou, Jiangsu, China

**Keywords:** Deinococcus radiodurans, pprI, radioresistance, acute radiation injury, electroporation, Pathology Section

## Abstract

PprI accelerates radiation-induced DNA damage repair via regulating the expression of DNA repair genes and enhances antioxidative enzyme activity in *Deinococcus radiodurans* after radiation. The main aim of our study was to determine whether the expression of *pprI* gene could fulfil its DNA repair function in eukaryotes and enhance the radioresistance of eukaryotic organism or not. In this study, we constructed pEGFP-c1-pprI eukaryotic expression vector and established a human lung epithelial cell line BEAS-2B with stable integration of *pprI* gene. We found that pprIexpression enhanced radioresistance of BEAS-2B cells, decreased γ-H2AX foci formation and apoptosis in irradiated BEAS-2B cells and alleviated radiation induced G2/M arrest of BEAS-2B cells. Moreover, we transferred pEGFP-c1-pprI vector into muscle of BALB/c mice by in vivo electroporation and studied the protective effect of prokaryotic *pprI* gene in irradiated mice. We found that pprI expression alleviated acute radiation induced hematopoietic system, lung, small intestine and testis damage and increased survival rate of irradiated mice via regulating Rad51 expression in different organs. These findings suggest that prokaryotic *pprI* gene expression in mammalian cells could enhance radioresistance *in vitro* and *in vivo*.

## INTRODUCTION

*Deinococcus radiodurans* (*D. radiodurans*) is one of the most radioresistance life-forms which have been found so far [1, 2]. In recent years, great attention was paid to *D. radiodurans* due to its dramatic capability to withstand the lethal and mutagenic effects of ionizing radiation, ultraviolet and other physical and chemical damages [3, 4]. The extremely radioresistant bacterium *D. radiodurans* possesses a rapid and efficient DNA damage response mechanism to survive lethal radiation damage [5, 6].

DNA repair is an essential process for cells to maintain their genomic stability [7, 8]. PprI (also called IrrE), a protein that is unique to the *Deinococcus-Thermus* family, has been identified as one of the essential proteins for the DNA damage response and repair processes [9, 10]. Inactivation of PprI causes the bacteria sensitive to various DNA damage. *PprI* gene serves as a general switch of DNA repair and protection pathways in *D. radiodurans* [10]. PprI accelerates radiation-induced DNA damage repair *via* regulating the expression of *recA*, *pprA* and other DNA repair genes and enhances the enzyme activities of catalase [10-12]. It is noteworthy that expression of *D. radiodurans PprI* gene enhances the radioresistance of *Escherichia coli* [12]. However, whether the expression of *D. radiodurans pprI* gene could fulfil its DNA repair function in eukaryotes and enhance the radioresistance of eukaryotes or not still remain elusive. *D. radiodurans* is a prokaryote and thus differs considerably from eukaryotes in gene composition, methods of protein expression, codon preference and so on. Moreover, PprI protein has no homologous analogue in mammalian cells. Interestingly, Geisler et al demonstrated that a eukaryotic recombinant protein production platform could be glycol-engineered with a bacterial gene which could be used to initiate sialic acid biosynthesis. The insect cells expressing this gene could produce sialylated N-glycoproteins without N-acetylmannosamine supplementation [13]. Sun et al explored the effects of the human immmunodeficiency virus-1/acquired immunodeficency syndrome (HIV-1/AIDS) trans-activator of transcription (Tat) protein on human rhabdomyosarcoma cellular responses to ionizing radiation and found that HIV-1 Tat protein sensitizes cells to ionizing radiation *via* depressing DNA repair and dysregulating cell cycle checkpoints [14]. We wondered whether the pprI gene could be expressed in mammalian cells and whether its expression have any effects on irradiated mammals. To date, there are no publications on this in the scientific literature.

In this study, we constructed pEGFP-c1-pprI eukaryotic expression vector and established a human lung epithelial cell line BEAS-2B with stable integration of *pprI* gene. We found that *pprI* expression enhanced radioresistance of BEAS-2B cells and decreased γ-H2AX foci formation in irradiated BEAS-2B cells. Moreover, we transferred pEGFP-c1-pprI vector into muscle of BALB/c mice by *in vivo* electroporation and studied the protective effect of prokaryotic *pprI* gene in irradiated mice. We found that *pprI* expression alleviated acute radiation induced hematopoietic system, lung, small intestine and testis damage and increased survival rate of irradiated mice by regulating Rad51 protein, a homologisation analogue of RecA in mammalian cells, expression level. These findings suggest that prokaryotic *pprI* gene expression in mammalian cells could enhance radioresistance *in vitro* and *in vivo*.

## RESULTS

### PprI expression in stable cell line

**Figure 1:**
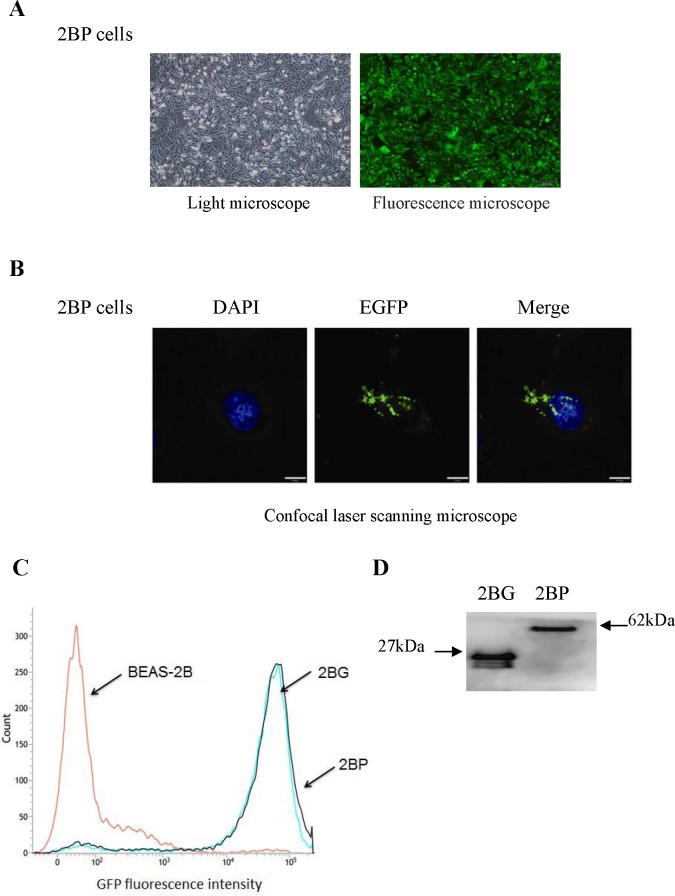
PprI expression in BEAS-2B cells with stable integration of *pprI* gene **A.** BEAS-2B cells with stable integration of *pprI* gene were established. The pictures are at 100× magnification. **B.** The localization of PprI protein in 2BP cells. Scale bar = 10 μm. **C.** Flow cytometric analysis of ROS formation in BEAS-2B cells. **D.** The fusion protein expression was detected by Western blotting.

DNA was isolated from the *D. radiodurans* wildtype strain R1 and *pprI* gene was amplified by PCR (Figure S1). The inserted sequence in recombinant vectors pEGFP-c1-pprI was sequenced (Figure S2), then compared with gene bank. The sequencing results showed that the amplified *pprI* gene was identical to the sequence in gene bank (Accession: AAF09762). Representative photos of BEAS-2B cells with stable integration of *pprI* gene in light microscope and in fluorescence microscope were shown in Figure 1A. It was shown in Figure 1B that PprI protein was in both cytoplasm and nucleus in the representative photos of BEAS-2B cells with stable integration of *pprI* gene in confocal laser scanning microscope. Moreover, GFP fluorescence intensity of BEAS-2B cells were detected by flow cytometer. The results showed that the fluorescence intensity of pEGFP-c1-pprI transfected cells (2BP group) and the negative control vector pEGFP-c1 transfected cells (2BG group) were significantly higher than the untransfected cells (2B group) (Figure 1C). To determine whether the 2BP cells could express PprI protein, we next detected the fusion protein expression using EGFP antibody by Western blotting. The results showed that the fusion protein (62 kDa) of EGFP (27 kDa) and PprI (35 kDa) was expressed in 2BP cells (Figure 1D).

### PprI expression enhanced radioresistance of BEAS-2B cells

**Figure 2:**
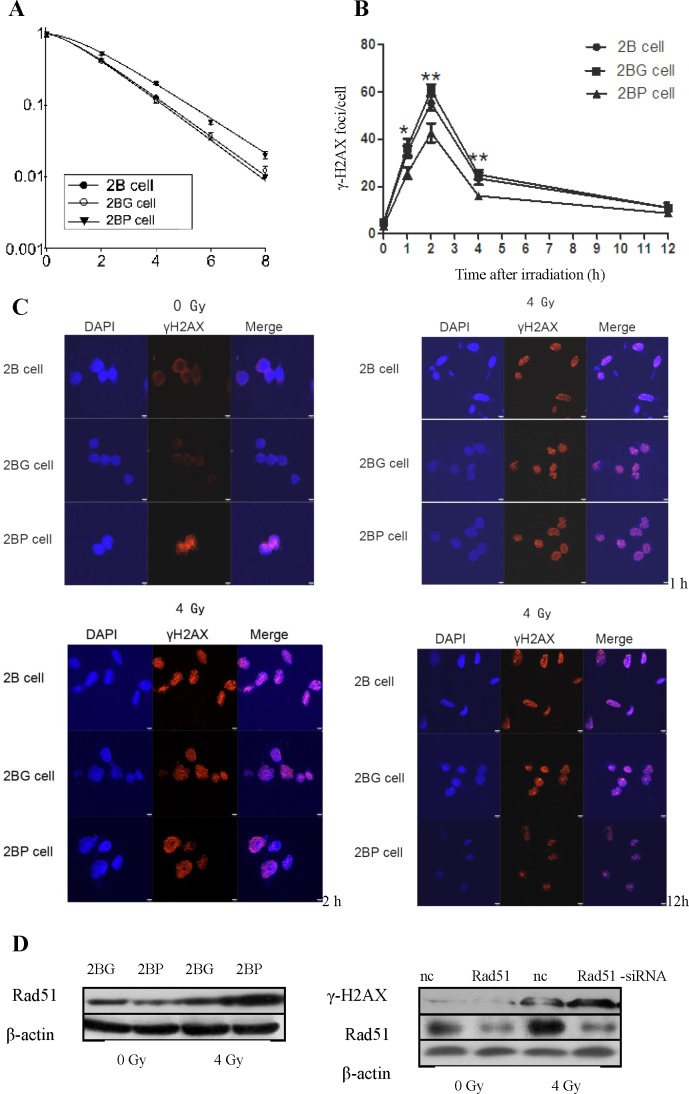
PprI expression enhanced radioresistance of BEAS-2B cells and decreased γ-H2AX foci formation in irradiated BEAS-2B cells **A.** Survival curves of BEAS-2B cells with stable integration of *pprI* gene. After exposure to 0, 2, 4, 6 and 8 Gy γ-ray irradiation, cell survival fractions were examined and the survival curves of cells were obtained from data fitting according to the linear quadratic model. Error bars indicate the standard error of the mean of three individual experiments. **B.** PprI expression decreased γ-H2AX foci formation in irradiated BEAS-2B cells. *P < 0.05, **P < 0.01 *vs* 2BG cells. **C.** Detection of γ-H2AX foci in irradiated BEAS-2B cells by immunofluorescence. **D.** PprI up-regulated Rad51 expression and Rad51 knockdown could increase γ-H2AX expression in irradiated 2BP cells.

After exposure to different doses γ-ray irradiation, the survival curves of BEAS-2B cells were obtained from data fitting according to the linear quadratic model (Figure 2A). It is clear that 2BP cells (D_0_ = 1.77 Gy, Dq = 1.28 Gy, *N* = 2.03, SF2 = 0.53) were more radioresistant than 2B cells (D_0_ = 1.57 Gy, Dq = 0.98 Gy, *N* = 1.70, SF2 = 0.43).

### PprI expression decreased γ-H2AX foci formation in irradiated BEAS-2B cells

γ-H2AX foci formation in BEAS-2B cells were detected after exposure to 4 Gy γ-ray irradiation. It was shown in Figure 2B and 2C that γ-H2AX foci in 2BP cells were significantly lower than that in 2B cells and 2BG cells 1, 2 and 4 h post-irradiation. These results indicated that PprI expression enhanced the DNA damage repair capacity in irradiated BEAS-2B cells. The eukaryotic protein Rad51 protein is highly homologous to RecA protein in terms of both structure and function, which plays a very important role in homologous recombination repair in *D. Radiodurans* [15, 16]. We next examined Rad51 expression by western blot in 2BG cells and 2BP cells 4 h post-irradiation. Compared with 2BG cells, Rad51 protein was significantly increased in 2BP cells after irradiation. Moreover, we investigated whether Rad51 knockdown could increase γ-H2AX expression in 2BP cells. After Rad51-siRNA transfection, Rad51 protein expression was significantly decreased, and γ-H2AX expression was significantly increased in 2BP cells 2 h post-irradiation (Figure 2D). These results indicated that Rad51 protein played an important role in PprI-induced radioresistance. We further investigated the possible mechanisms involved in the up-regulation of Rad51 expression, we analyzed Rad51 mRNA expression in 2BG cells and 2BP cells at various time point post-irradiation. Real-time RT-PCR analysis indicated that Rad51 mRNA expression level in 2BP cells showed no significant difference compared with that in 2BG cells (data not shown), suggesting that up-regulation of Rad51 protein in 2BP cells might be due to post-transcriptional, translational or post-translational regulation induced by PprI protein.

### PprI expression alleviated radiation induced G2/M arrest in BEAS-2B cells

**Figure 3:**
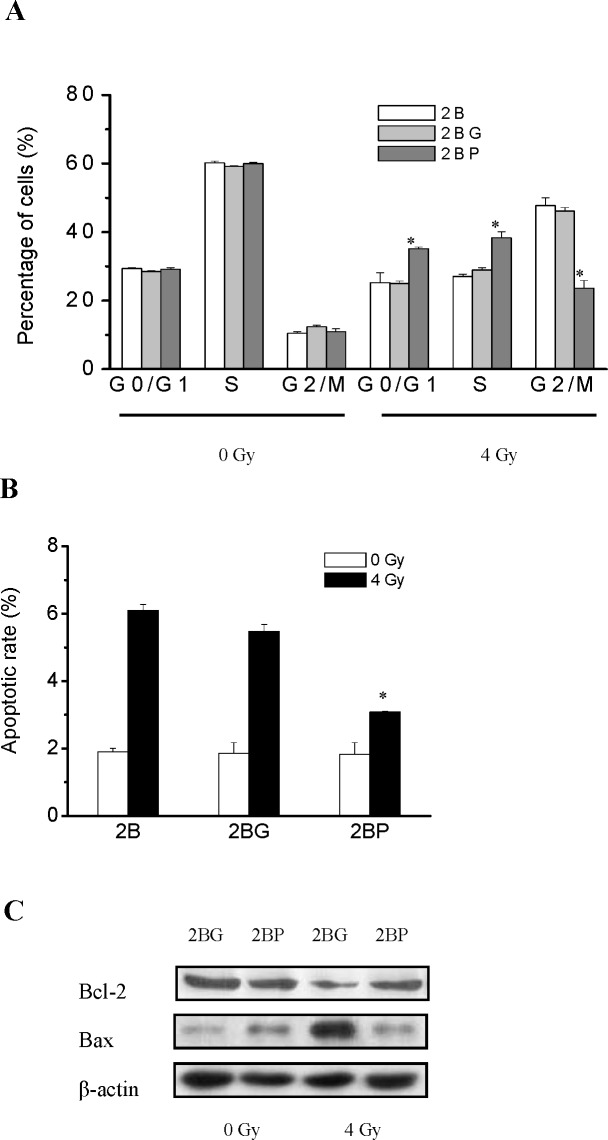
PprI expression alleviated radiation induced G2/M arrest and apoptosis in BEAS-2B cells **A.** Cell cycle of BEAS-2B cells were analyzed by flow cytometry 12 h post-irradiation. *P < 0.01 *vs* 2BG cells. **B.** Apoptosis of BEAS-2B cells were analyzed by flow cytometry 48 h post-irradiation. *P < 0.01 *vs* 2BG cells. **C.** Bcl-2 and Bax expression was examined using western blot 48 h after irradiation.

Cell cycle of BEAS-2B cells were analyzed by flow cytometry 12 h post-irradiation. After exposure to 4 Gy γ-ray irradiation, the percentage of G2/M phase in 2B, 2BG and 2BP cells were increased, while the percentage of S phase cells was significantly reduced. The percentage of G2/M phase in irradiated 2BP cells was significantly decreased compared with that in irradiated 2B and 2BG cells, while the percentage of G0/G1 and S phase cells were significantly increased (Figure 3A). These results suggested that PprI expression alleviated radiation induced G2/M arrest in BEAS-2B cells.

### PprI expression decreased radiation induced apoptosis in BEAS-2B cells

Apoptosis of BEAS-2B cells were analyzed by flow cytometry 48 h post-irradiation. After exposure to 4 Gy γ-ray irradiation, the apoptosis rate of 2B, 2BG and 2BP cells were increased. The apoptosis rate of irradiated 2BP cells was significantly decreased compared with that of irradiated 2B and 2BG cells (Figure 3B). The protein Bcl-2 plays an important role during the mitochondrial control of apoptosis. We analyzed the expression of apoptosis-related proteins (Bcl-2 and Bax) using western blots 48 h after irradiation. Compared to levels in 2BG cells, PprI appears to reduce the expression of the pro-apoptosis protein Bax and increase the concentration of anti-apoptosis protein Bcl-2 in 2BP cells (Figure 3C). These results suggested that PprI expression decreased radiation induced apoptosis in BEAS-2B cells.

### Fluorescence intensity in the muscle tissue of mice

The green fluorescence intensity in the local muscle tissue of mice under different conditions was shown in Figure S3. In a certain plasmid injection dose (50μg), the GFP fluorescence intensity of muscle tissue gradually increased as the electric field intensity increases and reached the maximum at a field strength of 200 v/cm, then the fluorescence intensity of GFP was decreased as the electric field intensity increases. On the other hand, in a certain electric field strength (200 v/cm), the GFP fluorescence intensity of muscle tissue gradually increased with the increase of plasmid injection dose and in doses of 50 μg reached the maximum. Subsequently, the fluorescence intensity of GFP was decreased with the increase of plasmid injection dose. Therefore, a plasmid injection dose of 50 μg/50 μL and a electric filed intensity of 200 v/cm were used for transfection in the subsequent study.

### PprI expression increased survival rate of irradiated mice

**Figure 4:**
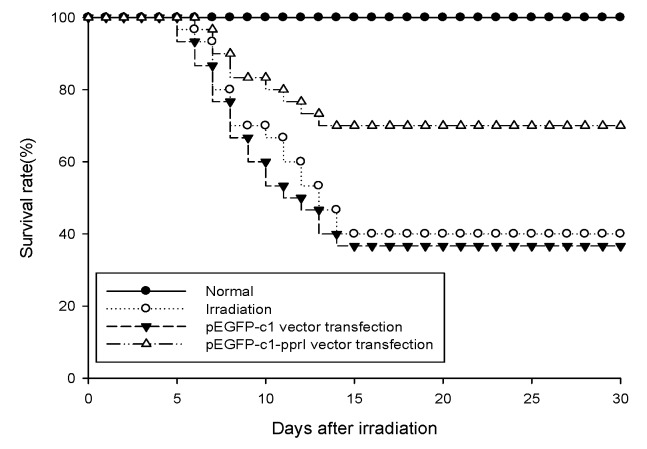
PprI expression increased survival rate of irradiated mice The protective effect against radiation *in vivo* of PprI expression was evaluated by survival rates of irradiated mice.

The protective effect against radiation *in vivo* of PprI expression was evaluated by survival rates of irradiated mice. The 30-day survival rates of mice after 6 Gy irradiation were shown in Figure 4. pEGFP-c1-pprI vector transfection group improved the survival rate by 30% compared with irradiated mice and improved the survival rate by 33.33% compared with pEGFP-c1 vector transfection group. These results suggested that PprI expression increased survival rate of irradiated mice.

### Effect of PprI expression on the peripheral blood cells of irradiated mice

**Figure 5:**
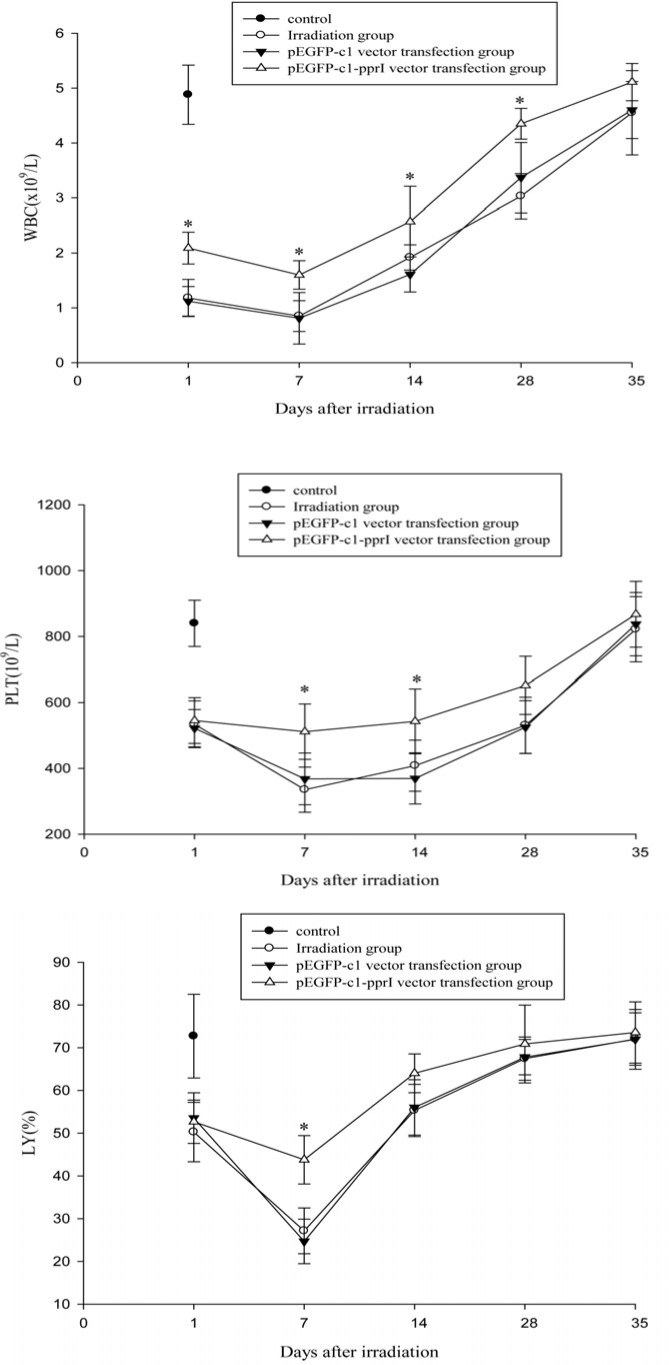
Effect of *pprI* expression on the peripheral blood cells of irradiated mice **A.** Changes of WBC counts in mice on Days 1, 7, 14, 28 and 35 after 4 Gy irradiation. *P < 0.05 *vs* pEGFP-c1 vector transfection group. **B.** Changes of platelet counts in mice on Days 1, 7, 14, 28 and 35 after 4 Gy irradiation. *P < 0.05 *vs* pEGFP-c1 vector transfection group. **C.** Changes of lymphocyte counts in mice on Days 1, 7, 14, 28 and 35 after 4 Gy irradiation. *P < 0.05 *vs* pEGFP-c1 vector transfection group.

Changes of WBC, platelet, and lymphocyte counts in mice on Days 1, 7, 14, 28 and 35 after 4 Gy irradiation were shown in Figure 5. Compared with irradiation group and pEGFP-c1 vector transfection group, pEGFP-c1-pprI vector transfection group showed significantly increased WBC counts on Days 1, 7, 14 and 28, platelet counts on Days 7 and 14 and lymphocyte percentage on Day 7.

### Effect of PprI expression on apoptosis of thymus, spleen and bone marrow cells of irradiated mice

**Figure 6:**
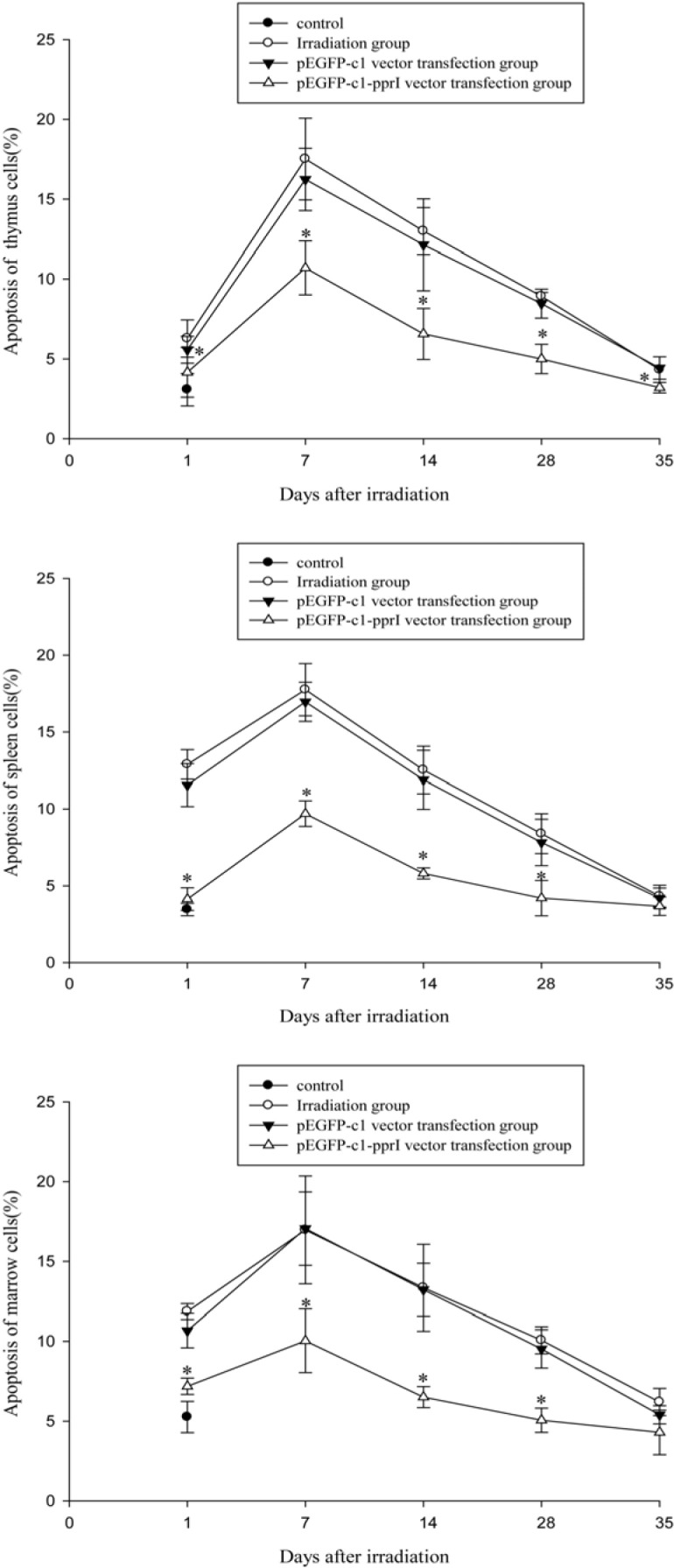
Effect of *pprI* expression on apoptosis of thymus, spleen and bone marrow cells of irradiated mice **A.** Changes of apoptosis rates of thymus cells in mice on Days 1, 7, 14, 28 and 35 after 4 Gy irradiation. *P < 0.05 *vs* pEGFP-c1 vector transfection group. **B.** Changes of apoptosis rates of spleen cells in mice on Days 1, 7, 14, 28 and 35 after 4 Gy irradiation. *P < 0.05 *vs* pEGFP-c1 vector transfection group. **C.** Changes of apoptosis rates of bone marrow cells in mice on Days 1, 7, 14, 28 and 35 after 4 Gy irradiation. *P < 0.05 *vs* pEGFP-c1 vector transfection group.

Changes of apoptosis rates of thymus, spleen and bone marrow cells in mice on Days 1, 7, 14, 28 and 35 after 4 Gy irradiation were shown in Figure 6. Apoptosis rates of the three kinds of cells were increased after irradiation, reached the maximum on the 7th days and then began to recover. Compared with irradiation group and pEGFP-c1 vector transfection group, pEGFP-c1-pprI vector transfection group showed significantly decreased apoptosis rates, demonstrating a radiation protective effect of the *pprI* gene.

### Overexpression of *pprI* gene reduced acute radiation induced lung toxicity in mice

**Figure 7:**
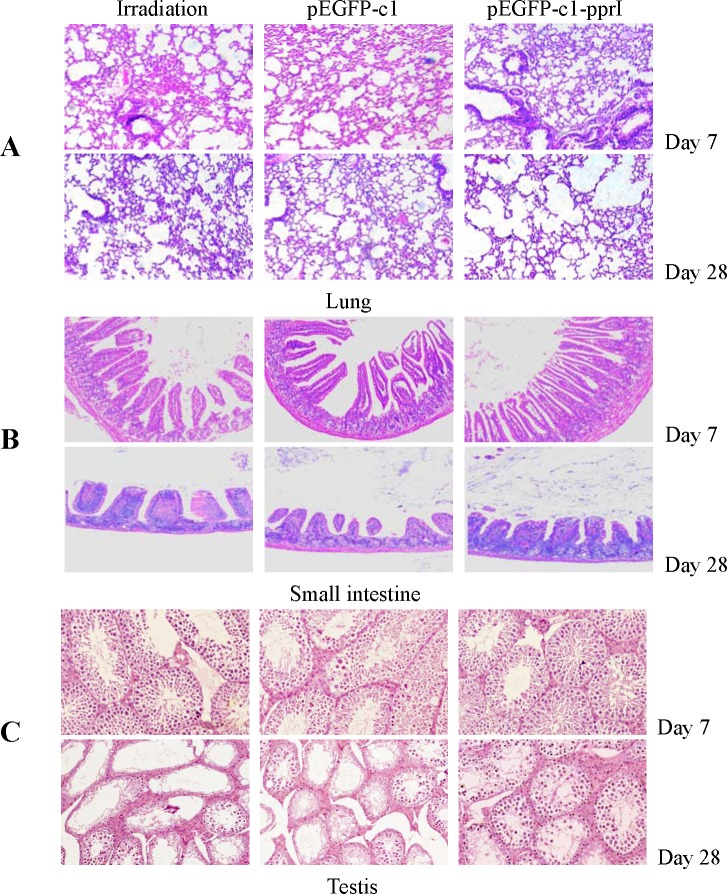
Overexpression of *pprI* reduced acute radiation induced damage in mice **A.** The histopathological changes in the lungs on Day 7 and Day 28 after irradiation (HE, 100×). **B.** The histopathological changes in the small intestines on Day 7 and Day 28 after irradiation (HE, 100×). **C.** The histopathological changes in the testis on Day 7 and Day 28 after irradiation (HE, 100×).

The histopathological changes in the lungs on Day 7 and Day 28 after irradiation were shown in Figure 7A. The lungs of mice in irradiation group and pEGFP-c1 vector transfection group exhibited severe vascular congestion of alveolar walls, erythrocytes extravasation and alveolar septa thickness by edema on Day 7 after irradiation, while the lungs of mice in pEGFP-c1-pprI vector transfection group showed mild hyperemia, edema and exudation. On Day 28 after irradiation, the lungs of mice in the irradiation group and pEGFP-c1 vector transfection group showed thickening and fibrosis of alveolar septa and epithelium and alveolar deformation by pressure, while the histopathological structure of lungs in pEGFP-c1-pprI vector transfection group basicly returned to normal. These results suggested that overexpression of *pprI* reduced acute radiation induced lung toxicity in mice.

### Overexpression of *pprI* gene reduced acute radiation induced small intestine damage in mice

The histopathological changes in the small intestines on Day 7 and Day 28 after irradiation were shown in Figure 7B. Intestinal villi exhibited serious damage, became sparse and stubby and basement membrane was broken on Day 7 after irradiation in the small intestines of mice in the irradiation group and pEGFP-c1 vector transfection group, while the damage in the small intestines of mice in pEGFP-c1-pprI vector transfection group was mild to moderate. A sparse intestinal villi and an incomplete basement membrane were still noted in the mice of irradiation group and pEGFP-c1 vector transfection group on Day 28 after irradiation, while the histopathological structure of the small intestine of mice in pEGFP-c1-pprI vector transfection group had recovered and almost returned to normal. These results suggested that overexpression of *pprI* reduced acute radiation induced small intestine damage in mice.

### Overexpression of *pprI* reduced acute radiation induced testis damage in mice

The histopathological changes in the testis on Day 7 and Day 28 after irradiation were shown in Figure 7C. The testis of mice in irradiation group and pEGFP-c1 vector transfection group exhibited significant atrophy and necrosis of the seminiferous tubules as well as degeneration and necrosis of spermatogonia and spermatocytes on Day 7 after irradiation, while the testis of mice in pEGFP-c1-pprI vector transfection group showed mild to moderate damage. The “blank area”, caused by necrosis of seminiferous epithelia cells, in the seminiferous tubules of mice in irradiation group and pEGFP-c1 vector transfection group still could be observed on Day 28 after irradiation while the testis of mice in pEGFP-c1-pprI vector transfection group made a full recovery. These results suggested that overexpression of *pprI* reduced acute radiation induced testis damage in mice.

### Mechanism of radiation protective effect of PprI expression *in vivo*

**Figure 8:**
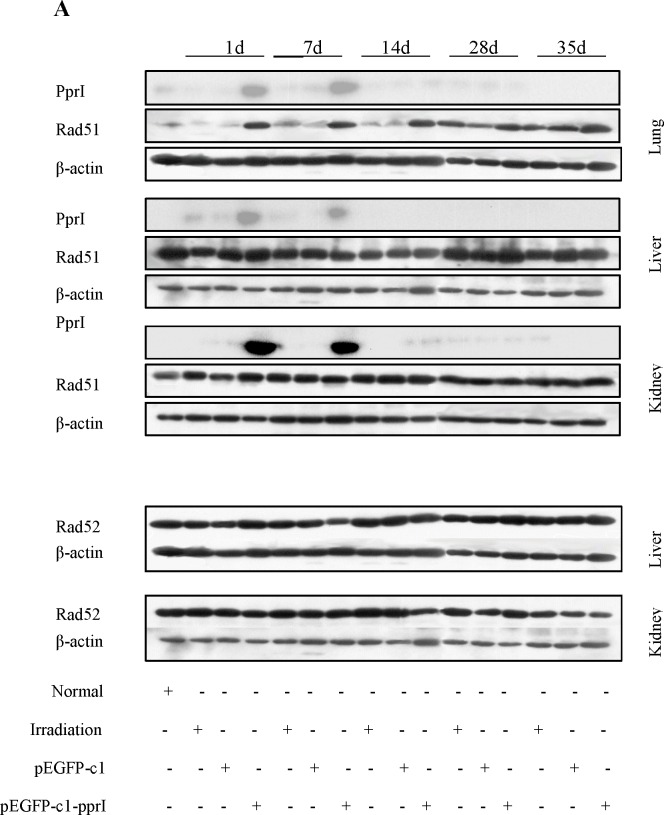

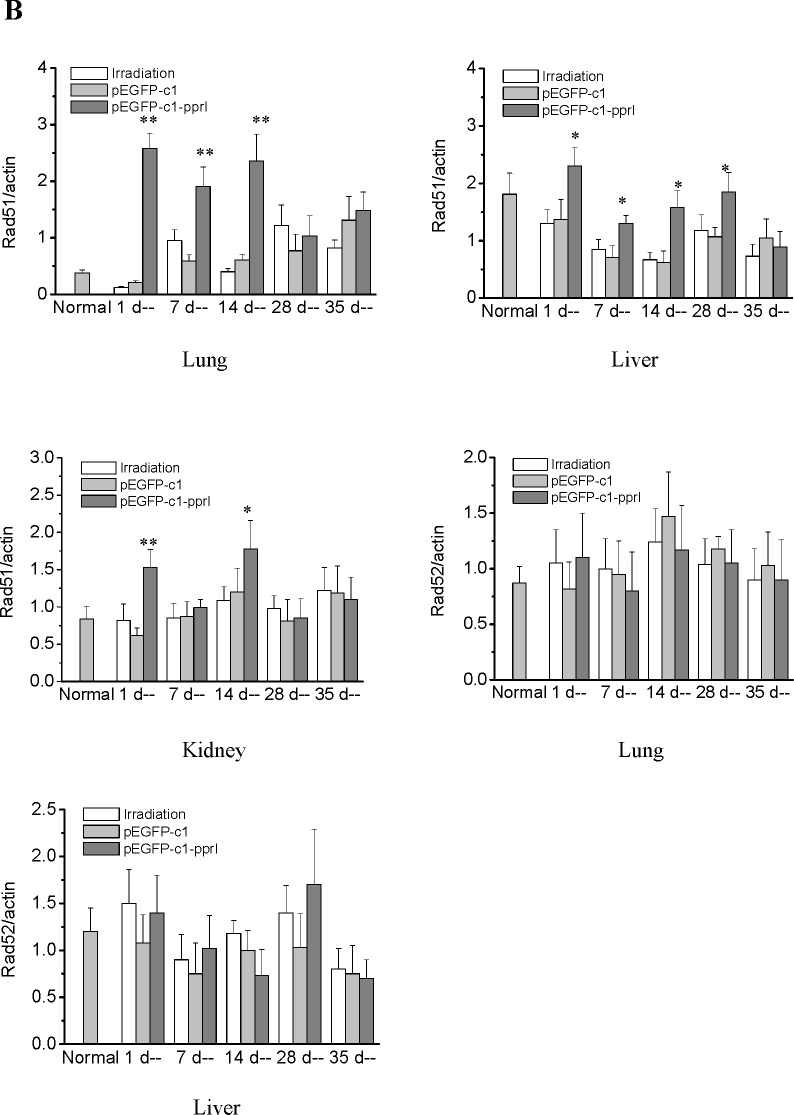
Mechanism of radiation protective effect of *pprI* expression *in vivo* **A.** Detection of PprI, Rad51 and Rad52 expression by western blot in the lung, liver and kidney of mice on Day 1, 7, 14, 28 and 35 after irradiation. **B.** Semi-quantitative data of Rad51 and Rad52 expression in the lung, liver and kidney of mice on Day 1, 7, 14, 28 and 35 after irradiation.

We examined the time course of PprI protein expression on Day 1, 7, 14, 28 and 35 after irradiation by detecting green fluorescence intensity in the local muscle tissue of mice in pEGFP-c1-pprI vector transfection group. The results showed that PprI protein was significantly expressed on Day 1 after irradiation, but it was not detectable on Day 7, 14, 28 and 35 after irradiation (data not shown). PprI protein was also examined by western blot in the lung, liver and kidney of mice. It was shown in Figure 8A that PprI protein was significantly expressed on Day 1 and 7 after irradiation, but it was not detectable later. Rad51 protein, a homologisation analogue of RecA in mammalian cells, might be regulated by PprI protein. We next examined Rad51 and its interacting protein, Rad52, expression by western blot in the lung, liver and kidney of mice on Day 1, 7, 14, 28 and 35 after irradiation [17, 18]. Compared with pEGFP-c1 vector transfection group, Rad51 protein was significantly increased in the lung on Day 1, 7 and 14 after irradiation, in the liver on Day 1, 7, 14 and 28 after irradiation and in the kidney on Day 1 and 14 after irradiation of mice in pEGFP-c1-pprI vector transfection group. Nevertheless, Rad52 protein showed no significant changes in the lung and liver of mice in pEGFP-c1-pprI vector transfection group compared with pEGFP-c1 vector transfection group on Day 1, 7, 14, 28 and 35 after irradiation (Figure 8A and 8B). These results suggested that the radiation protective effect of overexpression of *pprI* might at least in part result from the induced expression of Rad51, a 339-amino acid protein that plays a major role in homologous recombination of DNA during double strand break repair [19, 20], in different organs of mice.

## DISCUSSION

Ionizing radiation brings great benefits to human economic and life, at the same time a variety of nuclear and radiation accidents also continue to occur and cause acute radiation injury (ARI). In recent years, in response to the nuclear emergency and to improve the national nuclear safety level, studies on treatment and protection of ARI have become an important research field in the world.

*PprI* is the key gene of *Deinococcus radiodurans* in respond to DNA damage repair. PprI can bind to the promoter regions of *recA* and *pprA*, two genes important for DNA repair [21, 22]. Crystal structure suggests that pprI possesses three structure domains: a zinc peptidase-like domain, a helix-turn-helix motif and a GAF-like domain [23]. It was proposed that the state of PprI might change in response to DNA damage and its conformation might also be rearranged. The special constitution of the three domains in PprI indicates their synergistic effect on function and PprI is considered a generalist rather than a classic transcriptional regulator [22, 23]. Recently, Wang et al showed that the regulatory mechanism of PprI depended on its Mn(2+)-dependent protease activity toward DdrO, a transcription factor that suppresses DNA damage response (DDR) genes' expression and relieved the repression on many DNA repair genes including recA, ssB and ddrB. When DNA damage was fixed, PprI deactivated and DdrO reestablished the suppression of the DDR genes [24]. Here, we show *pprI* gene eukaryotic expression induced Rad51 protein, homologous to RecA protein in mammalian cells, *in vitro* and *in vivo*, in which the possible mechanisms involved is different from the transcriptional regulation involved in *D. Radiodurans* and might be post-transcriptional, translational or post-translational regulation by PprI protein, which is worthy of in-depth study.

In this study, pEGFP-c1-pprI eukaryotic expression vector was transferred into BALB/c mice by electroporation and the proper plasmid injection dose and electric field intensity were screened out according to the fluorescence intensity. *In vivo* electroporation is considered to be an effective transfection method and has been applied in many tissues and animal models [25-28]. Our studies confirmed that human cells and mice transfected with *pprI* eukaryotic expression vector exhibited a radioresistant phenotype, such as decreased radiation induced apoptosis and cell cycle arrest and enhanced DNA repair capacity.

High doses of ionizing radiation can cause severe ARI leading to multiple organ damage and a high mortality rate [29]. Though BALB/c mice were quite sensitive to radiation, our study showed that the mortality of BALB/c mice in *pprI* gene transfection group was significantly lower than irradiated group and empty vector transfection group, indicating that *pprI* gene transfer protected mice from ionizing radiation.

Hematopoietic and immune system are composed of bone marrow, thymus, spleen and lymph tissue, which are highly sensitive to ionizing radiation [30], therefore the changes of hematopoietic system caused by ionizing radiation can be used as an important index for the diagnosis and prognosis of ARI. Here, we observed that WBC counts, PLT counts, lymphocyte percentages decreased and apoptosis rates of thymocyte, spleen and bone marrow cells increased in different groups of mice after irradiation, while the changes in *pprI* gene transfection group was significantly lower than irradiated group and empty vector transfection group. The results suggested that the *pprI* gene improve the body's defense capability and accelerate the repair of radiation damage by regulating the immune and hematopoietic system.

ARI is often expressed as a multiple system damage, in addition to hematopoietic and immune function inhibition, radiation-induced lung, small intestine and testis damage are common pathological changes [31, 32]. After 4 Gy γ-ray irradiation, the mice in *pprI* gene transfection group showed milder damage and faster recovery compared with irradiated group and empty vector transfection group, indicating *pprI* gene accelerated the repair of tissue injury. Though the duration of PprI protein expression in the local muscle tissue was no more than 7 days, the induced expression of Rad51 lasted for 14 or 28 days in different organs, which might play an important role in the radiation protective effect of PprI.

In summary, *D. radiodurans pprI* gene transfection could improve the radiation resistance of human cells and mice, which provides valuable information for prokaryotic *pprI* gene in protection and therapy of mammalian ARI. The molecular mechanisms underlying the radiation protective effect of *pprI* in mammalian cells is an intriguing question that warrants further investigation.

## MATERIALS AND METHODS

### Bacterial strains, medium and culture conditions

The *D. radiodurans* wildtype strain R1, grown at 32°C in TGY broth (0.5% tryptone, 0.1% glucose, 0.3% yeast extract) with aeration, was kindly provided by Dr. Yuejin Hua in Key Laboratory of Chinese Ministry of Agriculture for Nuclear-Agricultural Science of Zhejiang University.

### *PprI* eukaryotic expression vectors construction

Full-length *pprI* gene was amplified from the wild type strain R1 genome by PCR using specific forward 5′-ATGCCCAGTGCCAACGTCAGCCCCCCTT-3′ and reverse 5′-TCACTGT GCAGCGTCCTGCGGCTCGTCC-3′ primers. The amplified DNA fragment was cloned into the pEGFP-c1 vectors with digestion by EcoR I and BamH I. The recombinant vectors pEGFP-c1-pprI were confirmed by the digestion analysis of restriction endonuclease and inserted sequences were verified by DNA sequencing.

### Cell culture and transfection

The non-tumorigenic human bronchial epithelial cell line BEAS-2B was purchased from the Type Culture Collection of the Chinese Academy of Sciences and cultured in DMEM/F12 medium (1:1, Hyclone) supplemented with 10% fetal bovine serum (FBS) (Invitrogen) in a humidified atmosphere containing 5% CO_2_ at 37°C. BEAS-2B cells were divided into three groups: pEGFP-c1-pprI transfected cells (2BP group), the negative control vector pEGFP-c1 transfected cells (2BG group) and untransfected cells taken as control (2B group). BEAS-2B cells were seeded in 6-well plates at 2.0×10^4^ cells/well and cultured to 80% confluence. Transfection was performed using LipofectAMINE (Gibco BRL, Gaithersburg, MD, USA) according to the manufacturer's instruction. Solution A was prepared by diluting 5 μg pEGFP-c1-pprI or pEGFP-c1 into 100 μl serum-free media. Solution B was prepared by diluting 10 μl LipofectAMINE into 100 μl serum-free media. The two solutions were mixed gently and incubated in room temperature for 30-45 minutes. The mixture and 800 μl serum-free media were added into each well. The cells were incubated at 37°C for 6 hours, and then the transfection media were replaced by fresh complete growth media.

### Generation of stable cell lines

After transfection, BEAS-2B cells with stable integration of *pprI* gene were selected using puromycin. After 3 weeks, single clones were analyzed for positive green fluorescent protein (GFP) signals [33]. The positive clones were expanded for additional testing.

### Western blot analysis of protein expression

Western blotting was performed using standard procedures. The following primary antibodies were used: rabbit polyclonal anti-Rad51, Rad52, Bcl-2 and Bax, mouse monoclonal anti-EGFP (Abcam, Cambridge, MA, USA) and rabbit monoclonal anti-β-actin (Santa Cruz Inc. California, USA). Experiments were repeated three times. The relative levels of protein expression were normalized against protein levels of an internal control gene, β-actin, performed in the same run.

### Clonogenic cell survival assay

BEAS-2B cells were irradiated with a cobalt-60 γ-radiation source (GWXJ80, Nuclear Power Institute of China, Chengdu, China) at a dose rate of 2 Gy/min at room temperature. After irradiation, a specific number of cells (100 for cells irradiated with 0 or 2 Gy, 200 for 4 Gy and 2000 for 6 and 8 Gy) were plated in petri dishes in triplicate for clonogenic assay. Then the cells were incubated for 10 days. Colonies were fixed by 37% formaldehyde solution and stained with crystal violet and colonies of more than 50 cells were counted. Furthermore, the cell survival fraction was counted out and the cell survival curve was drafted by the standard model, S = 1-(1-e^−D/D0^)^N^ (S, cell survival fraction; D, radiation dose; e, the bottom of the natural logarithm; D_0_, the mean death dose; N, extrapolate number).

### Immunofluorescence

BEAS-2B cells were stained with primary antibody for γ-H2AX (Epitomics) and slides were incubated for 1 h with Alexa-488-conjugated anti-rabbit IgG for visualization of foci. Luorescence analyses were performed with a DM 6000 B microscope (Leica, Wetzlar, Germany). Every microscope slide was counted at least three times by two blinded observers independently. In order to get the x-ray induced γ-H2AX-foci (so-called excess γ-H2AX-foci) we subtracted the absolute γ-H2AX-foci before irradiation (so-called background foci) from the absolute γ-H2AX-foci after exposure.

### Rad51-siRNA design and transfection

The cDNA sequence of Homo sapiens gene Rad51 was obtained from GenBank (NM_133487). The siRNA target design tools from Ambion were used to design Rad51-siRNA. Rad51-siRNA was designed and synthesized as follows (Sangon Inc. Shanghai, China): sense: 5′-CCAGCUCCUUUAUCAAGCATT-3′, antisense: 5′-UGCUUGAUAAA GGAGCUGGGT-3′. BEAS-2B cells with stable integration of pprI gene were plated 24 h prior to transfection. Cells were transfected in 6-well plates by use of Lipofectamine RNAiMAX (Invitrogen, Carlsbad, CA, USA). Rad51-siRNA and negative control siRNA (nc-siRNA) were used at 100 nM final concentration.

### Flow cytometric analysis of cell cycle

BEAS-2B cells were harvested 48 h after transfection and fixed overnight with 70% ethanol at 4°C, followed by resuspension in 500 μL of PBS. After addition of 10 μL RNase (10 mg/mL), cells were left for 30 minutes at 37°C and stained with 10 μL propidium iodide (1 mg/mL). Cellular DNA content was determined on a flow cytometer Beckton Dickinson (*BD*) *FACScan* (*BD* Biosciences, San Jose, CA) with an exciting wavelength of 488 nm. The relative proportion of cells in the G_0_/G_1_, S and G_2_/M phases of the cell cycle were determined by flow cytometry data.

### Flow cytometric analysis of apoptosis

Quantification of apoptotic cells was performed according to the Annexin-V-PE/7-AAD Apoptosis Detection Kit manufacturer instructions (KeyGen Biotech. Nanjing, China). Analyses were performed by a flow cytometer (BD FACScan). Phycoerythrin (PE)-positive and 7-amino-actinomycin D (7-AAD) -negative cells were regarded as apoptotic cells.

### Mice

Male BALB/c mice (6-8 weeks old, weighting 16-20 g) were purchased from SLAC Laboratory Animal Co., Ltd. (Shanghai, China). In order to optimize transfection dose and electric field intensity, the mice were randomly divided into 8 groups of 3 mice each: plasmid injection (50 μg); plasmid injection (50 μg) + electric field intensity (100 v/cm); plasmid injection (50 μg) + electric field intensity (150 v/cm); plasmid injection (50 μg) + electric field intensity (200 v/cm); plasmid injection (50 μg) + electric field intensity (250 v/cm); plasmid injection (40 μg) + electric field intensity (200 v/cm); plasmid injection (60 μg) + electric field intensity (200 v/cm); plasmid injection (70 μg) + electric field intensity (200 v/cm). The rest mice were randomly divided into the mortality rate observation group (40 mice) and experimental group (80 mice). Then the mice in mortality rate observation group were randomly divided into four groups: normal group (unirradiated), irradiation group, pEGFP-c1 vector transfection group and pEGFP-c1-pprI vector transfection group, with 10 mice in each group. The mice in experimental group was randomly divided into control group (5 mice), irradiation group (25 mice), pEGFP-c1 vector transfection group (25 mice) and pEGFP-c1-pprI vector transfection group (25 mice).

### *In vivo* electroporation

Different doses of endotoxin free plasmid pEGFP-c1 (1 μg/μL) or pEGFP-c1-pprI (1 μg/μL) were injected into anterolateral muscle of mice hind leg. One minute after the injection, the muscle tissues at the site of injection were given different electric field intensity stimulation (100, 150, 200 and 250 v/cm, 50 ms, 1Hz, 8 electric pulses) by *in vivo* gene transfection apparatus (ECM830, BTX company, American). Mice were sacrificed 24 h after transfection. The muscle tissues at the injection site were taken and the fluorescence intensity of GFP was observed by fluorescence microscopy. This study was performed at a facility accredited by the Association for the Assessment and Accreditation of Laboratory Animal Care (AAALAC) with approval from an Institutional Animal Care and Use Committee (IACUC).

### Irradiation

The mice in the mortality rate observation group and experimental group were exposed to a dose of 6 and 4 Gy of ^60^Co γ-ray total body irradiation (GWXJ80) at a dose rate of 2 Gy/min, respectively.

### Detection of peripheral hemogram of mice

Mice were sacrificed 1, 7, 14, 28 and 35 d after irradiation. Blood was taken from mice and put into anticoagulant tubes. WBC counts, PLT counts and lymphocyte percentages were analyzed by CELL-DYN 3700 blood cell analyzer (Abbott Laboratories Ltd, USA).

### Detection of apoptosis rates of thymocyte, spleen and bone marrow cells

Thymus, spleen and bone marrow were taken respectively for preparing monoplast suspension. Quantification of apoptotic cells was performed according to the Annexin V-FITC Apoptosis Detection Kit manufacturer instructions (KeyGen Biotech. Nanjing, China). Analyses were performed by a flow cytometer (BD FACScan). AnnexinV-positive and propidium iodide (PI) -negative cells were regarded as apoptotic cells.

### Histopathological examination

Lungs, small intestines and testis biopsy specimens from the mice in the experimental group were fixed in formalin and embedded in paraffin. Sections were cut, placed on glass slides, stained with Hematoxylin and eosin (HE) and observed under a light microscope (Nikon, Tokyo, Japan).

### Statistical analysis

Results were expressed as means ± standard deviations (SD). The data was analyzed using one-way analysis of variance on SPSS 16.0 and the group means were compared by LSD Test. *P* < 0.05 was considered significant.

## SUPPLEMENTARY MATERIAL FIGURES


