# Supplementary material for: Deinococcus radiodurans pprI expression enhances the radioresistance of eukaryotes
Source: Oncotarget. 2016 Mar 16;7(13):15339–55. doi: 10.18632/oncotarget.8137 (PMC4941245; doi:10.18632/oncotarget.8137)
Supplement: Supplementary file 1 [file oncotarget-07-15339-s001.pdf]

Deinococcus radiodurans pprI expression enhances the radioresistance of eukaryotes

Supplementary Material

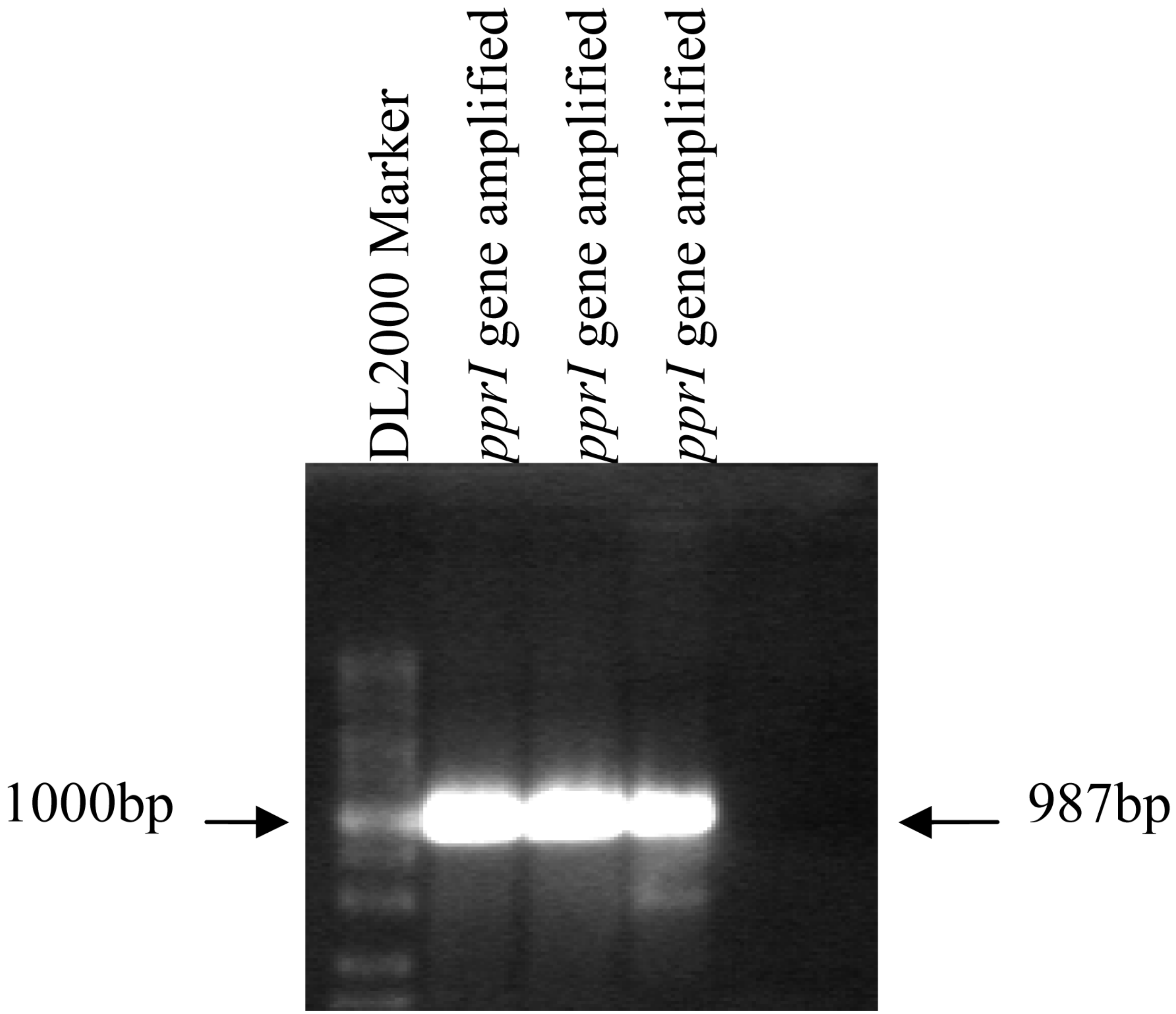

Fig. S1. *PprI* gene was amplified by PCR.

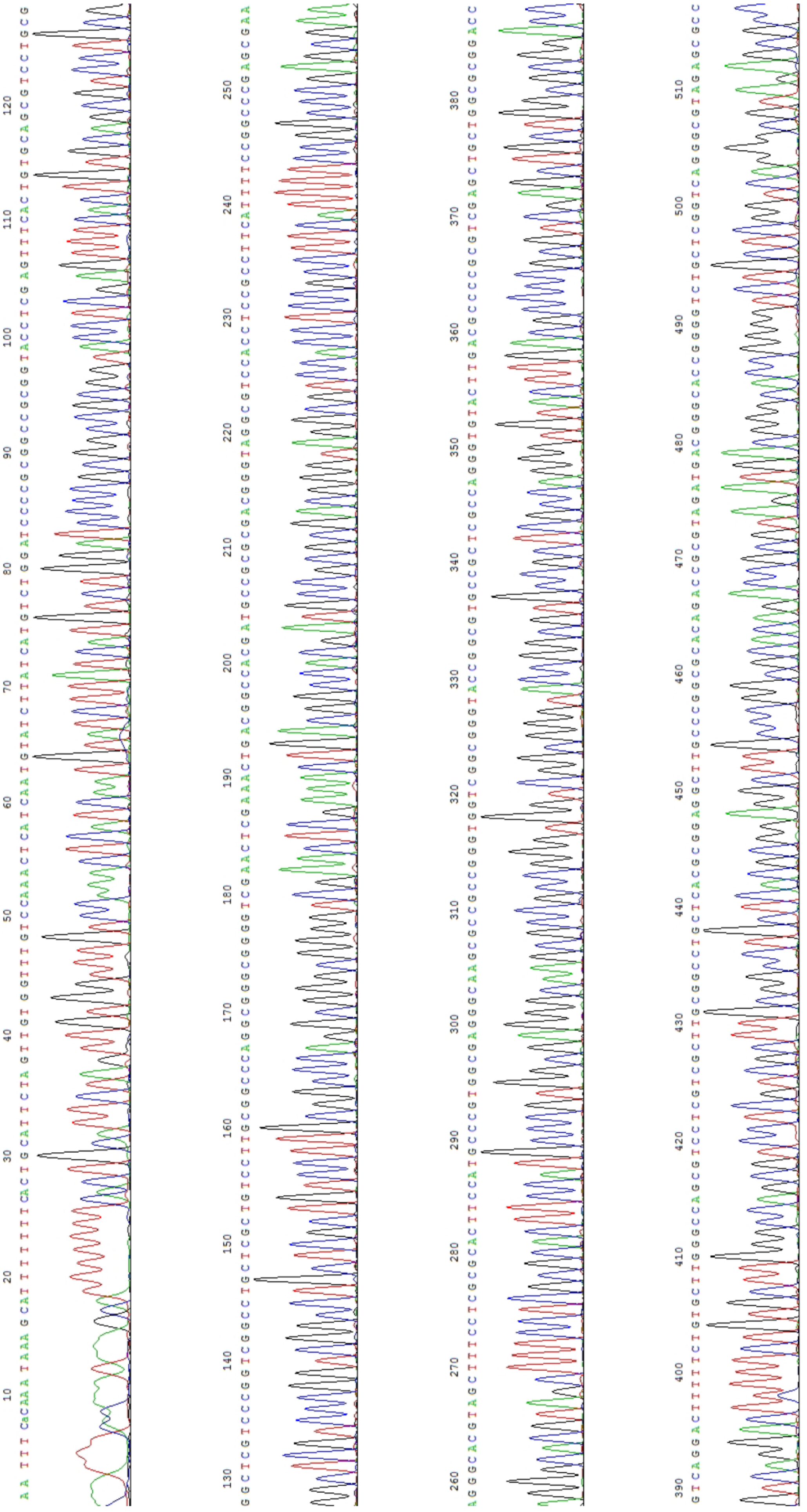

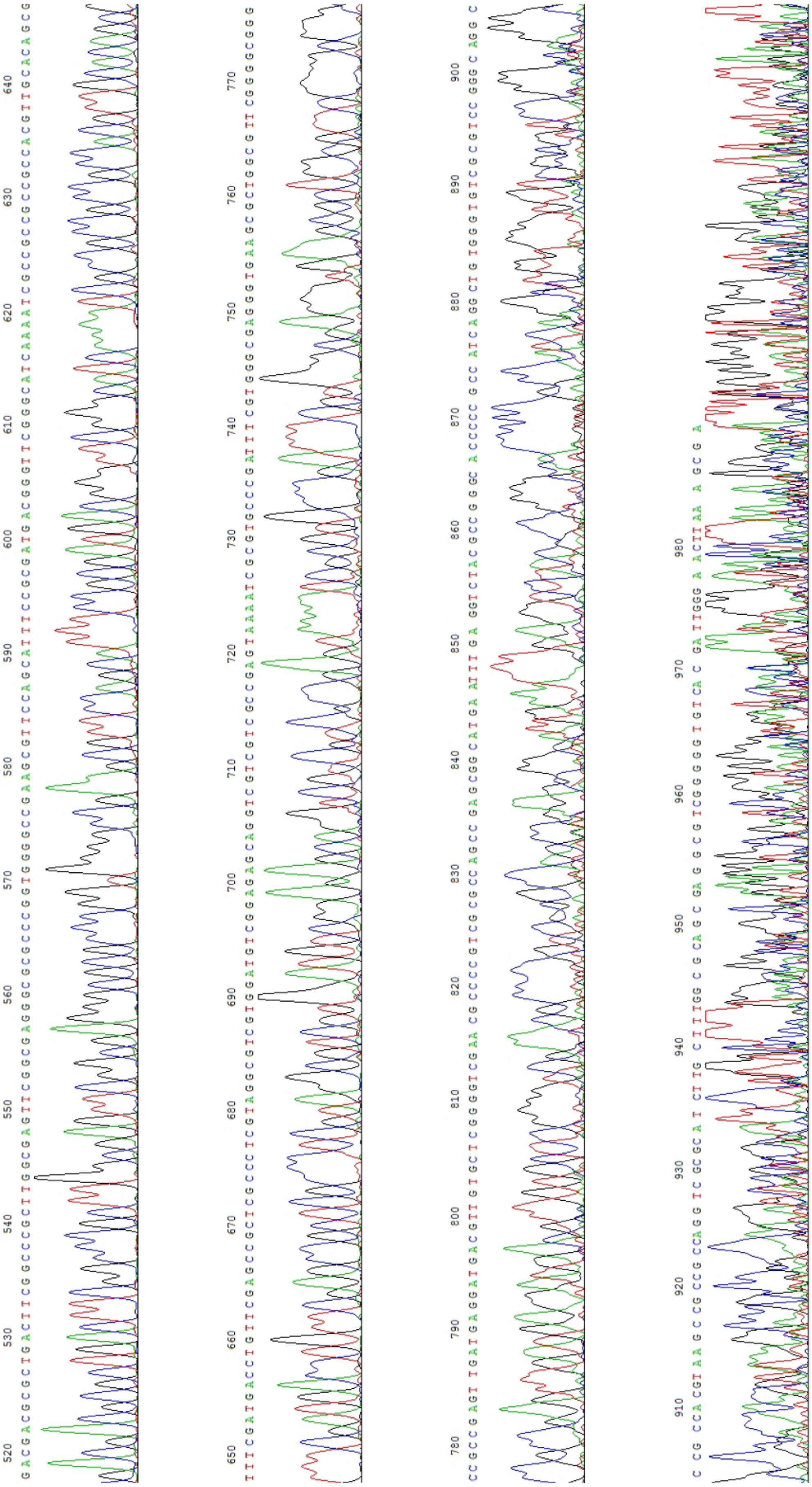

Fig. S2. The inserted sequence in recombinant vectors pEGFP-c1-*pprI* was sequenced.

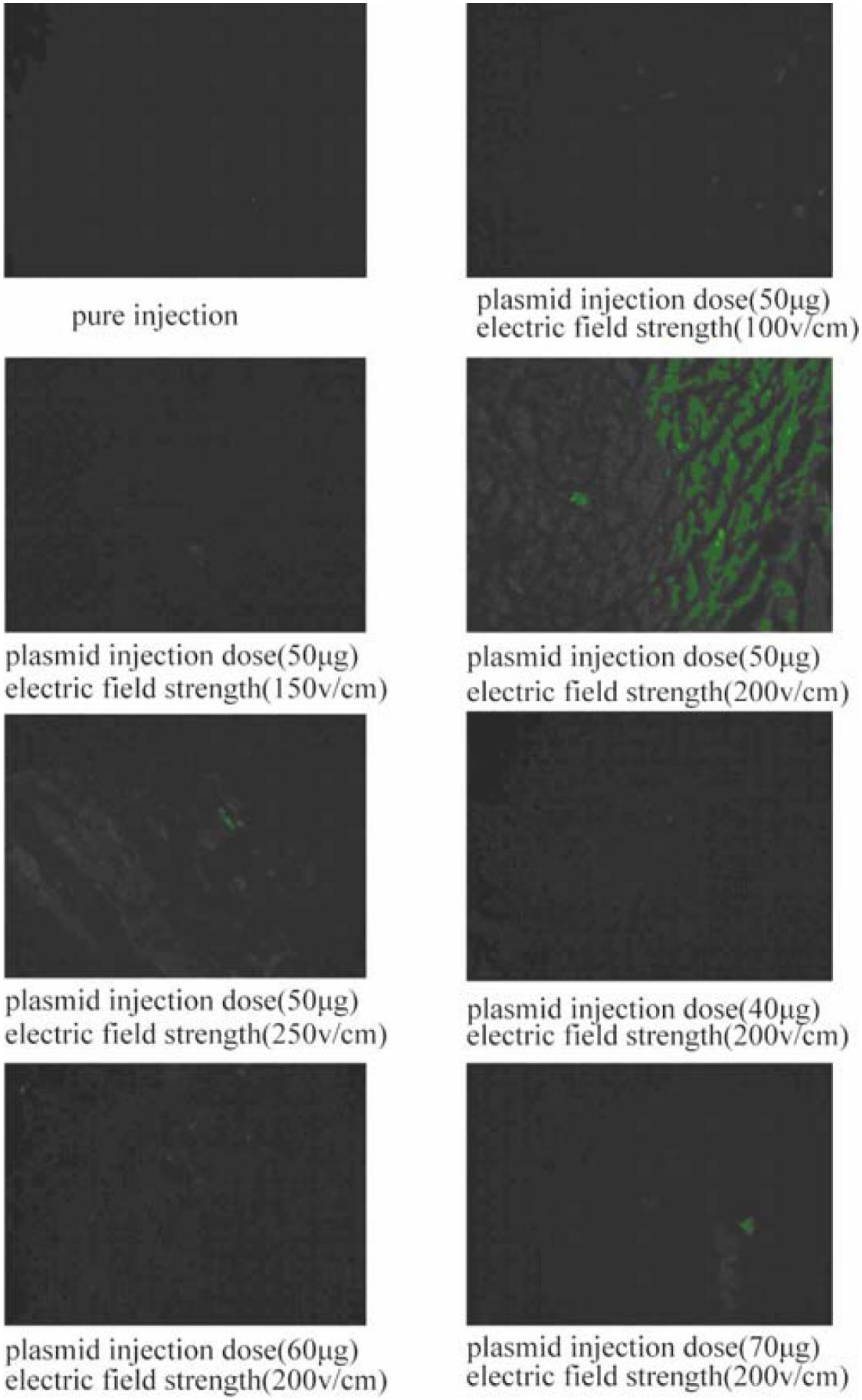

Fig. S3. The green fluorescence intensity in the local muscle tissue of mice under different conditions.
